# Topological abnormality of structural covariance network in MRI-negative frontal lobe epilepsy

**DOI:** 10.3389/fnins.2023.1136110

**Published:** 2023-05-05

**Authors:** Yin Liu, Quanji Li, Dali Yi, Junhong Duan, Qingxia Zhang, Yunchen Huang, Haibo He, Yunjie Liao, Zhi Song, Lingling Deng, Wei Wang, Ding Liu

**Affiliations:** ^1^Department of Radiology, The Third Xiangya Hospital, Central South University, Changsha, China; ^2^Department of Neurology, The Third Xiangya Hospital, Central South University, Changsha, China; ^3^Department of Radiology, The Second Affiliated Hospital, University of South China, Hengyang, China

**Keywords:** graph theory, structural covariance network, cortical thickness, frontal lobe epilepsy, default mode network (DMN)

## Abstract

**Background:**

Frontal lobe epilepsy (FLE) is the second most common type of focal epilepsy, however, imaging studies of FLE have been far less than Temporal lobe epilepsy (TLE) and the structural findings were not consistent in previous literature.

**Object:**

Investigate the changes in cortical thickness in patients with FLE and the alteration of the structural covariance networks (SCNs) of cortical thickness with graph-theory.

**Method:**

Thirty patients with FLE (18 males/12 females; 28.33 ± 11.81 years) and 27 demographically matched controls (15 males/12 females; 29.22 ± 9.73 years) were included in this study with high-resolution structural brain MRI scans. The cortical thickness was calculated, and structural covariance network (SCN) of cortical thickness were reconstructed using 68 × 68 matrix and analyzed with graph-theory approach.

**Result:**

Cortical thickness was not significantly different between two groups, but path length and node betweenness were significantly increased in patients with FLE, and the regional network alterations were significantly changed in right precentral gyrus and right temporal pole (FDR corrected, *p* < 0.05). Comparing to HC group, network hubs were decreased and shifted away from frontal lobe.

**Conclusion:**

The topological properties of cortical thickness covariance network were significantly altered in patients with FLE, even without obvious surface-based morphological damage. Graph-theory based SCN analysis may provide sensitive neuroanatomical biomarkers for FLE.

## 1. Introduction

Frontal lobe epilepsy (FLE) is the second common type of focal epilepsy behind temporal lobe epilepsy, accounting for ~20%–30% of sufferers (Manford et al., 1996). As the largest lobe of neocortex, the highly interconnected nature of the frontal lobe allows for a quick and widespread propagation of epileptic activity to the other brain regions, which may give rise to the perplexing clinical and electrophysiological finding of FLE. A variety of semiologies are common for FLE patients, such unilateral clonic seizures, tonic asymmetric seizures with preserved consciousness, hypermotor seizures, and secondary generalized seizures. The ambiguity in localization and lateralization of deficits of EEG is well known, even false negative EEG is not uncommon (Beleza and Pinho, 2011). Seizures in FLE are mostly likely to be associated with multi-cognitive defects and motor-related abnormality networks (Kellinghaus and Lüders, 2004; Beleza and Pinho, 2011), and the treatment outcome is disappointing, as only 20%–30% of patients achieve seizure freedom with medication (Regesta and Tanganelli, 1999). Of all patients with refractory focal epilepsies referred to epilepsy surgery, 25% have FLE, and only 30%–50% achieve seizure freedom with surgery (Bagla and Skidmore, 2011). As epileptogenic zone may be subtle and not obvious in routine MRI examination (Bonini et al., 2014), it presents challenges in FLE patients with ambiguous EEG pattern, and calls for better clarification of the underlying neuroanatomic characteristic of FLE, especially for patients with normal routine MRI examination.

As a brain network disorder, epilepsy has been widely studied using quantitative neuroimaging data, which supported that epileptogenic network are involved in the generation and expression of seizures, and to the maintenance of the disorder (Spencer, 2002). In contrast to make low-level regional and connectional alterations with conventional approach, graph-theory analysis provides a correlational framework to reveal the persistent functional-trophic cross-talk, maturational inter-change, as well as common developmental and pathological influences (Bullmore and Sporns, 2012; Alexander-Bloch et al., 2013; Bernhardt et al., 2013), and can be used for various modalities of neuroimaging data. Normal topological network is characterized by high clustering coefficients and short average path lengths. According to previous graph-theory based fMRI and diffusional MRI (dMRI) studies (Vaessen et al., 2013, 2014; Gleichgerrcht et al., 2015; Zhou et al., 2019; Lin et al., 2020; Togo et al., 2022), rearrangement of global and local topological parameters has been found in patients of focal epilepsy (including FLE and TLE), such as diminished network strength, clustering coefficient, path length, and global efficiency, both within and beyond the epileptogenic zone.

Unlike fMRI and dMRI, structural T1-weighted images is a standard component of every clinical imaging protocol with short acquisition time. These images are generally unaffected by distortion and signal drop out artifacts in orbitofrontal and temporo-basal regions which often occur in echo-planar functional and diffusion MRI sequences (Bernhardt et al., 2013; Gleichgerrcht et al., 2015). Graph theory based structural covariance network (SCN) of cortical thickness directly seeds from cortical gray matter regions in a high-resolution space, which is not limited by the imaging voxels but by the sampling density of the points on the cortical mesh (Hosseini et al., 2012; Alexander-Bloch et al., 2013). SCN analysis has been applied to investigate in various central nervous system disorders (Hosseini et al., 2012; Singh et al., 2013), and considered a promising tool in investigating the brain network alterations in epilepsy (Li et al., 2020), but it was rarely used in FLE patients. In order to investigate the topological property alteration of cortical thickness in FLE, SCN analysis was performed between a group of clinically diagnosed FLE patients with negative MRI and a age-, gender-, education-matched healthy control (HC) group in the present study.

## 2. Patients and methods

### 2.1. Patients

Thirty FLE patients (18 males, age range = 16–62 years; average age ± standard deviation = 28.33 years; standard deviation = 11.81 years) were recruited from Department of Neurology, the Third Xiangya Hospital, Central South University. All patients were diagnosed by senior neurologists based on comprehensive evaluation of clinical history, 24-h video-EEG recording, ictal semiology, routine MRI examination according to the International League Against Epilepsy (ILAE) guidelines (Engel, 2001). No structural abnormalities can be identified responsible for these FLE patients through the routine MRI examination, such as malformation, tumor, reactive gliosis, and other epileptogenic lesions. All patients underwent 24-h (overnight including the sleep period) scalp video-EEG recordings (EEG-1200C, Nihon Kohden, Tokyo, Japan). For EEG, 16 electrodes were distributed according to 10–20 international standard system, and the sampling rate was set at 256 Hz. All patients received antiepileptic drug (AED) treatments with regular out-patient follow-up. The detailed demographic information and the clinical characteristics of FLE patients can be seen in Table 1. A total of 27 age-, gender- and education-matched healthy volunteers were also recruited as controls (15 males, age range = 18–60 years, average age ± standard deviation = 29.22 years; standard deviation = 9.73 years). Written consent forms of all FLE patients and controls were obtained. The study protocol was approved by the Ethics Committee of the Third Xiangya Hospital, Central South University.

**Table 1:** Demographic, clinical, and neuropsychological test difference between FLE and HC group.

Clinical characteristic	FLE patients (*n* = 30)	HC (*n* = 27)	*p* value
Age at examination/year^a^	28.33 (11.81)	29.22 (9.73)	0.76
Gender/male^b^	18 (60%)	15 (56%)	0.73
Eduacation/year^a^	12.47 (2.73)	13.04 (3.31)	0.48
Disease duration/year^a^	8.50 (9.64)		
Localization (left/right/bilateral/unknown)	3/5/21/1		
Seizure frequency (grade 0/1/2/3)	9/13/1/7		
Medication (mono/multi medication)	24/6		

a Data in average (standard deviation).

b Data in number (percent).

Duration of illness, epileptic seizure types, initial and follow-up EEGs, medication protocol, the follow-up seizure attacks after treatment were also collected for all patients. The National Hospital Seizure Severity Scale (NHS3) which contains seven seizure-related factors on a score of 1 to 27 was used to assess the severity of epilepsy (O'Donoghue et al., 1996). The seizure attack frequency after treatment for each patient was evaluated according to International League Against Epilepsy (ILAE) scale (Wieser et al., 2001).

### 2.2. Image acquisition

MRI data were acquired using a 3.0 T MRI scanner (Ingenia, Philips Medical Systems, Netherlands) with a 15-channel receiver array head coil at the Department of Radiology, Third Xiangya Hospital, Central South University. The participants were instructed to lie quietly with their eyes closed but remain awake, and to avoid specific thoughts during scanning. To improve image quality, earplugs were used to attenuate scanner noise, and foam pads were applied to minimize head movements.

Structural images were acquired with a three-dimensional turbo fast echo (3D-TFE) T1WI sequence with high resolution as follows: repetition time (TR)/echo time (TE) = 9.1/4.5 ms; slices = 170; thickness = 1 mm; gap = 0 mm; flip angle (FA) = 8°; acquisition matrix = 256 × 256; field of view (FOV) = 240 mm × 240 mm.

### 2.3. Data preprocessing

The cortical thickness (CT) was determined using CAT12^1^ and SPM12^2^ based on the MATLAB 2014a operating environment (MathWorks, Natick, MA, USA). Briefly, the T1-weighted images underwent tissue segmentation to estimate white matter distance. Local maxima were then projected to other grey matter voxels by using a neighbor relationship described by the white matter distance. These values equal cortical thickness. Topological correction was performed through an approach based on spherical harmonics (Fischl and Dale, 2000). After preprocessing and visual checks for artefacts, all scans passed through the automated quality check promoted in the manual of CAT12. No participant was excluded by the automated quality check protocol. The brain was parcellated into 68 cortical regions using the Desikan-Killiany atlas (Desikan et al., 2006) and the mean cortical thickness was calculated for each region. The individual map of CT was smoothed with a Gaussian filter with a full-width at half-maximum of 15 mm to statistical analysis.

### 2.4. Cortical thickness structural covariance networks construction

**Figure 1:**
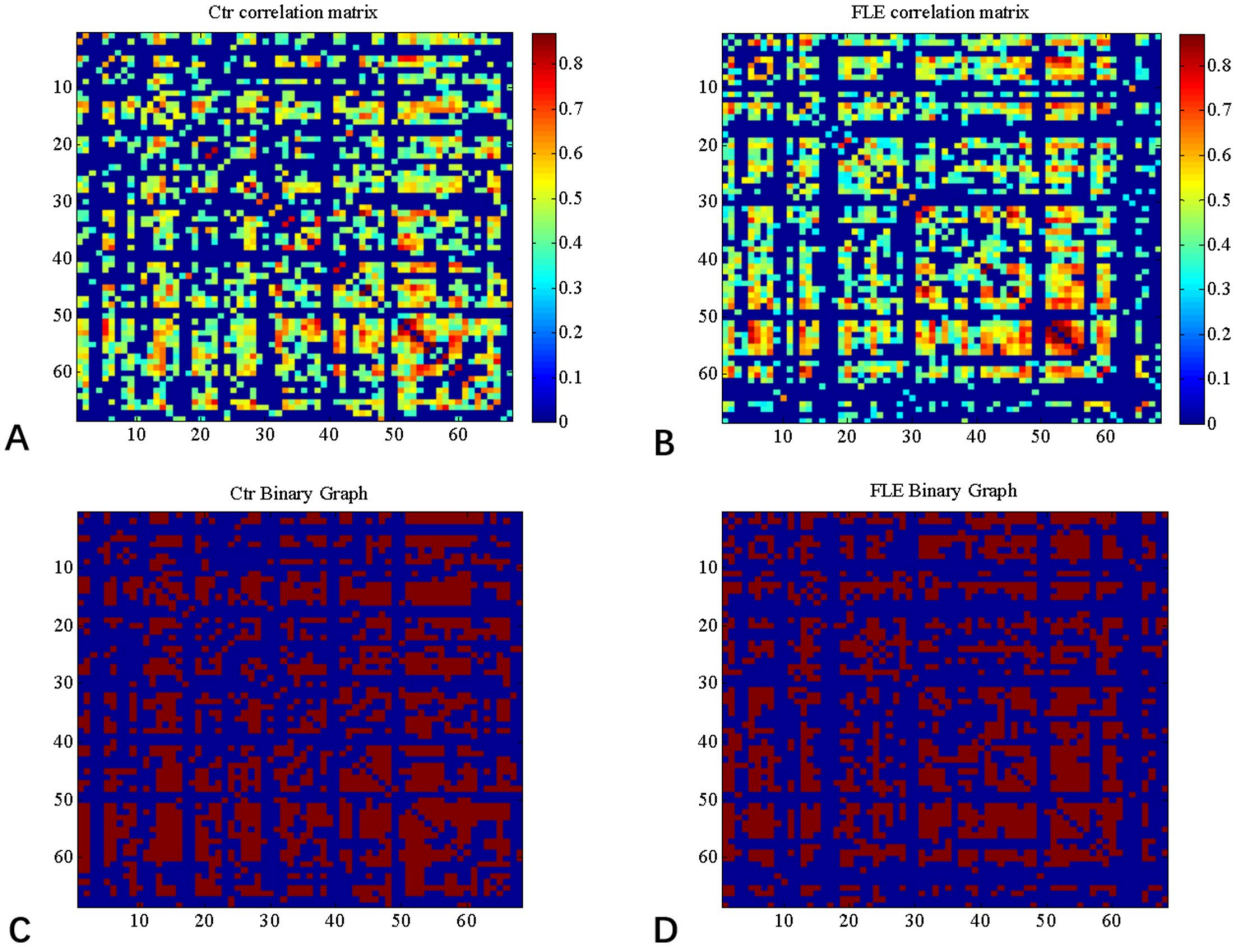
Correlation matrices and adjacency matrices with 68 × 68 for healthy controls (Ctrl) and frontal lobe epilepsy (FLE) patients. Correlation matrices for HC **(A)** and FLE patients **(B)** and binary adjacency matrices at the minimum density (0.38) for HC **(C)** and FLE patients **(D)**. Correlation matrices show the Pearson correlation coefficient between any two regions of the network and the color bar denotes the absolute value of the Pearson correlation coefficient and represents the strength of the connections.

Graph Analysis Toolbox (GAT) was used to construct the SCNs based on CT (Hosseini et al., 2012). A linear regression analysis was conducted for each cortical region to adjust the effect of age and gender, so that the corrected cortical thickness was obtained to construct SCN. According to the Desikan–Killiany Atlas (Desikan et al., 2006), a 68 × 68 correlation matrix was established for each group by calculating Pearson correlation coefficients between interregional corrected CT values. Thereafter, the correlation matrix was converted into a binary adjacency matrix by thresholding correlation coefficients into values of 1 or 0 (Figure 1). Here, these thresholds were defined as a range of network densities varying from 0.38 to 0.5 (increments of 0.02), which ensured that FLE and HC SCNs had the same number of nodes and edges at each density. The minimum density (0.38) was determined to ensure that the networks were not fragmented for both groups. For density above 0.5 the networks approached random configuration (Humphries et al., 2006; Singh et al., 2013). Inter-group differences of network topologies were compared across the range.

### 2.5. Global and regional network analyses

According to the definitions of these parameters in previous studies (He et al., 2008; Rubinov and Sporns, 2010), a series of global and regional network parameters was calculated to characterize the topological properties of the SCNs. Global network parameters included normalized clustering coefficient, normalized path length, and small-world index. Briefly, the human brain can be regarded as a small-world network that has the highest clustering coefficient (Cp) and shortest path length (Lp). Cp of a node pertains the number of existing connections linking the adjacent nodes divided by all their possible connections. The Cp of a network is the average of clustering coefficients across all nodes in a network, which represents network segregation. The shortest path length (Lp) is equal to the minimum number of edges that connect two nodes. The Lp of a network pertains to the average shortest path length involving all node pairs in the network, which represents network integration. The normalized clustering coefficient (gamma) and normalized path length (lamda) were calculated, respectively, by comparing the CP and Lp to the mean Cp and mean Lp of 1,000 random network (Maslov and Sneppen, 2002).

The nodal characteristics of the CT structural covariance network were examined and the alteration of regional network between groups were analyzed. Which included that the nodal local efficiency, nodal clustering coefficient and nodal betweenness centrality. The node local efficiency reflects the connection degree between a node and other nodes, representing the communication efficiency of the node. Nodal betweenness centrality is an important index which is defined as the number of shortest paths between any two nodes in the network that pass through a given node (Rubinov and Sporns, 2010). The graph index is used to detect important functional or anatomical connections. The quantified nodal local efficiency, clustering coefficient, and betweenness centrality were, respectively, normalized by the average network local efficiency, clustering coefficient, and betweenness centrality. Inter-group differences of these normalized region network parameters were compared.

### 2.6. Network hubs

Hubs are the most globally connected regions in the brain and are essential for coordinating brain function through the connectivity with numerous brain regions. Hubs play a central role in integrating diverse information sources and supporting fast information communication with minimal energy cost. In our study, the criteria for defining hub is that the node’s betweenness was at least 2 standard deviation higher than the mean network betweenness centrality (Hosseini et al., 2012).

### 2.7. Statistical analysis

Clinical data analysis was completed using IBM SPSS 26.0. Chi-square tests were used to compare categorical variables. Student’s *t*-test or the Mann–Whitney U-test were used to compare continuous variables between two groups.

For the CT maps, general linear modeling, installed in the CAT toolbox, was used to perform vertex-wise group inference on the smoothed cortical surfaces. To calculate significance of the differences in SCN parameters between groups, we analyzed the network parameters both at Dmin and across the density range (0.38–0.5 with an interval of 0.02) using area under the curve (AUC). A non-parametric permutation test (1,000 repetitions) was used to investigate the statistical significance of the difference in global and regional network parameters. The comparison of the global and regional parameters between groups was completed with the GAT toolbox with the result corrected by *p* < 0.05 with false discovery rate (FDR) considered to be statistically significant.

## 3. Results

### 3.1. Demographic and clinical characteristics

The demographic and clinical information of FLE and HC groups were listed in Table 1. All the patients were complex-partial seizure with secondary generalized tonic–clonic attack, with typical frontal lobe epilepsy characteristic such as hypermotion, dominance of attack during sleep. The average duration of disease was 8.50 (SD = 9.64). The NHS3 score for the patients ranged from 2 to 18, with the average ± SD of 9.67 ± 4.14.

### 3.2. Between-group comparison of CT

Comparing to HC group, only the cortical thickness of left postcentral gyrus was decreased in FLE group (*p* < 0.001,uncorrected), but the difference was not significant after FDR correction.

### 3.3. Inter-group differences in global network metrics

**Figure 2:**
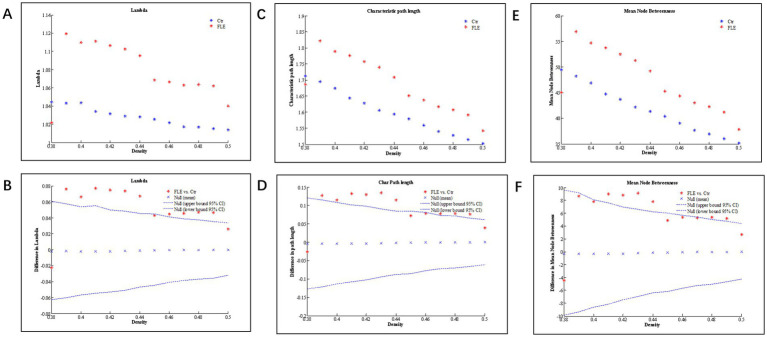
Changes and between-group differences of normalized path length (**A,B**; Lamda), characteristic path length **(C,D)**, and mean node betweenness **(E,F)** as a function of network density in healthy control (Ctrl) and frontal lobe epilepsy (FLE) groups. For between-group differences **(B,D,E)**, Except for a few densities, there are significant difference between two groups, as indicated by dots lying outside the 95% confidence intervals (dashed lines) (*p* < 0.05 after FDR correction).

Changes and between-group differences in global network parameters were significant between two groups at densities ranging from 0.38 to 0.50, as shown in Figure 2. Compared to HC group, both the characteristic and normalized path length of FLE group were significantly longer, and the mean node betweenness was also significantly higher in FLE group. The global and local efficiency, clustering coefficient (Gamma), and small-world index (Sigma) were lower in FLE, but they did not reach statistical significance (data not shown).

### 3.4. Inter-group differences in regional network metrics

**Figure 3:**
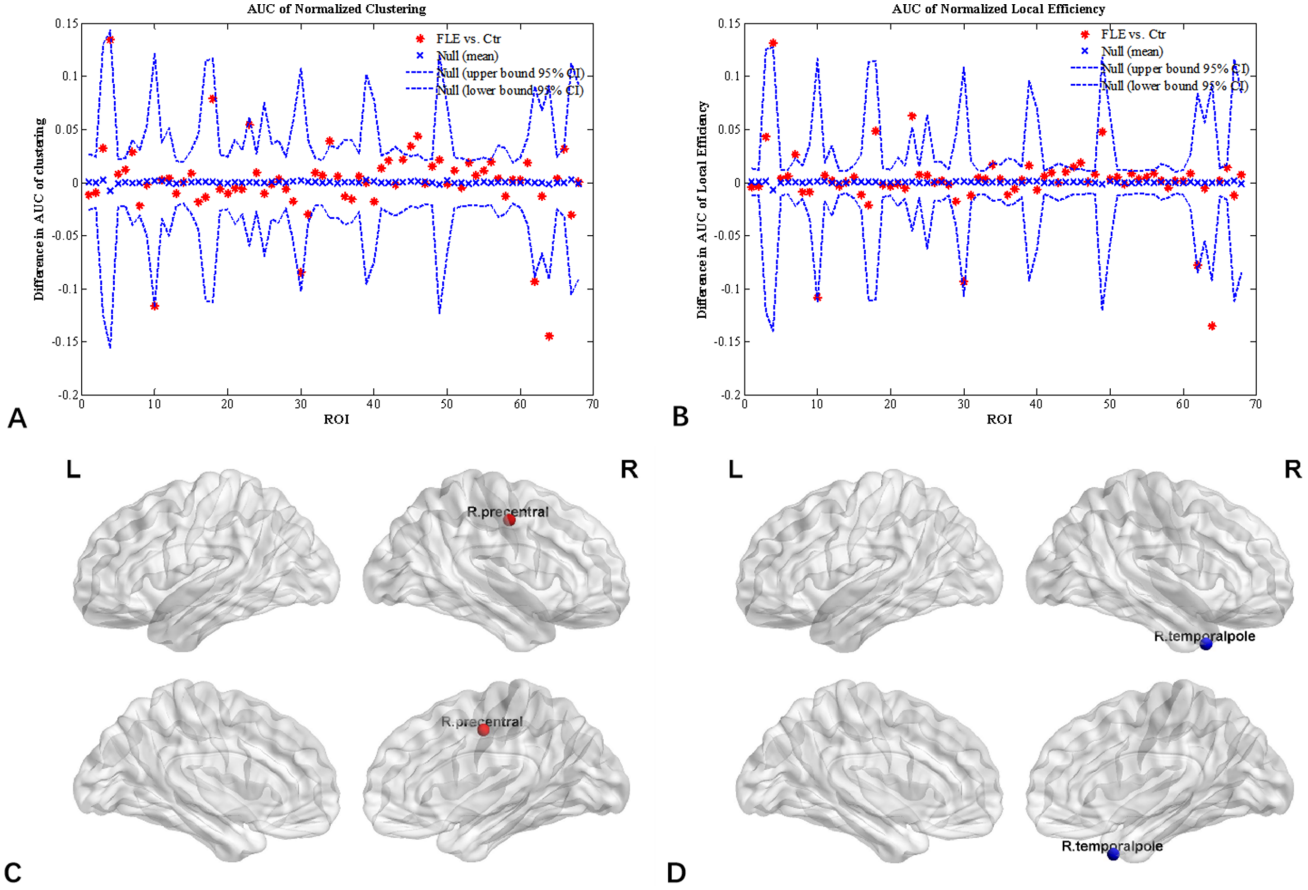
Regional network parameter difference between HC and FLE groups. Between-group differences of normalized clustering coefficient **(A)** and normalized local efficiency **(B)** across a range of network densities was shown. The red * lying outside of the confidence intervals indicates regions different between the two groups in this density, and cortical regions survived FDR correction (p < 0.05) were right precentral gyrus **(C)** with a increased clustering coefficient and local efficiency, and right temporal pole **(D)** with decreased clustering coefficient and local efficiency in FLE patients compared with HCs.

Inter-group differences in regional network metrics of normalized clustering and local efficiency were shown in Figure 3. Compared to HC group, both the normalized clustering and local efficiency of right precentral gyrus were significantly higher, and that of right temporal pole were significantly lower in FLE (*p* < 0.05 with FDR correction).

### 3.5. Network hubs

**Figure 4:**
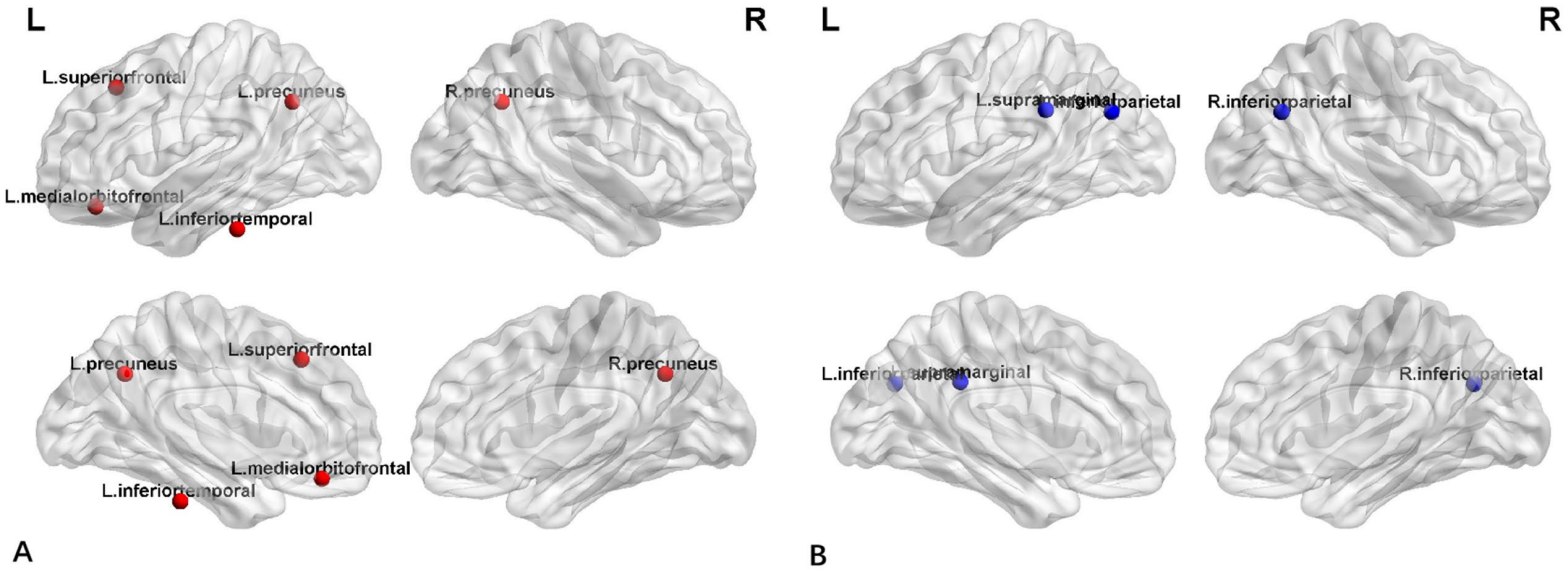
The distribution of network hubs in HCs **(A)** and FLE patients **(B)**.

Figure 4 displayed group-specific hubs for HC and FLE groups. Hubs determined for HC group network included left inferior temporal gyrus, left medial orbito-frontal gyrus, bilateral precuneus gyri, and superior frontal gyrus. Hub number for FLE group was reduced, which included left inferior parietal gyrus, right inferior parietal gyrus, and left supramarginal gyrus.

## 4. Discussions

This study investigated the topological property alteration of cortical thickness based SCN in FLE patients without structural abnormality on conventional MR. Both the global and regional network parameters were significantly different between FLE and HC groups. The results revealed that the structural network properties were significantly changed in MRI-negative FLE patients, suggesting higher sensitivity of graph-theory based analysis in detecting the neurobiological injury in FLE patients. To the best of our knowledge, the present study reported for the first time the cortical-thickness related topological property alteration in MRI-negative FLE patients and it may be a valuable tool in future clinical practice.

The structural alterations of FLE were not consistent in previous studies. In the study of Widjaja et al. (2011), widespread cortical thinning was found, while in other studies, there was no significant difference in cortical /GM volume between FLE and HC (Lu et al., 2022). In the present study, cortical thickness was not significantly different between the two groups. However, our study demonstrated that FLE patients had significantly different topological change for both global and regional network parameters, though both groups showed a small-world topology. Consistent with previous functional graph-theory based neuroimaging studies of epilepsy (Vaessen et al., 2013), the path length and node betweenness were significantly increased in FLE, indicating the global network topology was altered toward a regularized pattern. However, variations in structural network were also found. In the study of Vaessen et al. (2013), the structural path length and clustering remained normal in children with FLE, structural modularity was different between two groups and it was increased with stronger cognitive impairment. Thus modularity may be associated with cognitive status, which was not included in present study and warrants further investigation. In addition to methodological approach difference (e.g., different atlases and morphometric features), patient selection variation may also contribute to the discrepancy. For the study of Vaessen et al. (2013, 2014) and Widjaja et al. (2011), the patients recruited were children, but the patients in the present study were adults older than 14 years old. As cortical structure should be closely associate with age, and the variation during development and maturation of frontal lobe may be huge even in normal population (Silver et al., 2021; Herring et al., 2022), future studies with larger sample size is warranted to clarify the influence of age.

In FLE, the clinical symptoms of these seizures are variable and dependent on the brain regions, which may include peri-rolandic, supplementary sensorimotor area, dorsolateral frontal, orbitofrontal, anterior frontopolar, opercular, and cingulate types (Bagla and Skidmore, 2011). But most FLE patients usually are recognized as multi-cognitive defects and motor-related networks defects (Kellinghaus and Lüders, 2004; Beleza and Pinho, 2011). In the present study, all the patients were clinically diagnosed FLE, with the prominent seizure characteristics of hypermotor attacks and secondary generalized clonic–tonic seizure. This is in agreement with previous report on FLE (Bagla and Skidmore, 2011; Bonini et al., 2014) and we speculated that motor-related network abnormality should be found. In agreement with this speculation, both normalized clustering and local efficiency of right precentral gyrus were significantly increased, indicating an abnormal increase in network segregation in sensorimotor network. Similar findings were reported in previous study of FLE (Woodward et al., 2014).

Connections between temporal lobe and sensorimotor cortex have been repeatedly reported in previous neuroimaging studies of MRI-negative TLE patients, especially for right MTS to influence ipislateral thalamus and temporal pole (Coan et al., 2014; Roggenhofer et al., 2020). In addition to TLE, temporal pole and precentral gyrus involvement is also reported for patients with GTCS (Liu et al., 2017; Li et al., 2020). Thus, it is not surprising that regional topological parameters were also compromised for right temporal pole in the study. As a seizure is typically the result of the networks that are recruited or traveled by the epileptiform discharges, therefore seizure-onset localization can be misled by clinical manifestations that may arise from recruited networks that are remotely located from the ictal source. The decreased clustering and local efficiency of right temporal pole may reflect the intrinsic network pattern of fronto-limbic system.

Similarly, network hub analysis showed that there were significant difference between FLE and HC groups. In HC group, the hubs were evenly distributed around the neocortex, including bilateral precuneus, left inferior temporal gyrus and left medial orbitofrontal gyrus, which was consistent with DMN network hubs (Zhou et al., 2019). However, for FLE patients, the hubs were reduced and shifted posterior and none was found in frontal lobes. The hubs identified in FLE group, the inferior parietal lobule which is usually closely connected with precuneus (Togo et al., 2022). Together with supramarginal gyrus, IPL is a region implicated in a diverse range of higher cognitive functions and may be associated with multiple brain networks, including DMN, Frontoparietal control network, and cingulo-opercular network (Igelström and Graziano, 2017). This hub alteration pattern may reflect the DMN abnormality in FLE, which was also reported in previous fMRI studies of epilepsy (Lin et al., 2020; Togo et al., 2022). The mechanism was unknown, but it may result from the interrupted small-world properties within frontal lobe in FLE, as noted by the disappearance of network hub in frontal lobe in FLE.

In addition, the hubs found in FLE, were among regions of reduced cortical thickness reported by Widjaja et al. (2011), in a study of FLE children. Similarly, in the functional connectivity study of FLE patients by Wu et al. (2019), increased ALFF in the precuneus of FLE patient, despite their response to antiepileptic medication, and these were the hubs for HC group in the present study. Inferior parietal lobules were also found to be affected in patients with hyperkinetic seizures (Sasagawa et al., 2021). The clinical significance of the network hub alteration remains unknown and warrants further investigation.

The present study has several limitations. Firstly, the FLE patients were clinically diagnosed, which was based on seizure semiology and EEG; while magneto encephalography, FDG-PET, and invasive intracranial monitoring were unavailable for the patients. Thus the possibility of multiple origin other than frontal lobe could not be completely excluded. In addition, most of the patients could not be lateralized, thus it is not clear whether the laterality affected the cortical thickness and brain volume distribution. Also, the sample size was relatively small with various illness duration, and the medicine used was not the same among subjects. These would have potential effects on the topological results, and future studies with larger sample size and better homogeneity of FLE patients may provide further insights. Secondly, cross-sectional design was used in the present study which brings difficulty to get a causal conclusion. Longitudinal design may be needed in the future to further confirm our finding and assess whether the changes of network graph properties is the consequence of seizures.

In summary, the present study investigated topological properties of cortical thickness covariance network alteration in patients with FLE using the graph theory method. Both global and regional network parameters were significantly different between patients with FLE and normal controls, despite the surface-based cortical thickness was not significantly different between two groups. These results indicated that graph-theory based structural covariance network analysis may provide clues to reveal the structural alterations in MRI-negative FLE.

## Data availability statement

The raw data supporting the conclusions of this article will be made available by the authors, without undue reservation.

## Ethics statement

The studies involving human participants were reviewed and approved by the Ethics Committee of the Third Xiangya Hospital, Central South University. The patients/participants provided their written informed consent to participate in this study.

## Author contributions

YiL, WW, DL, and ZS designed the study. QL, DY, QZ, JD, YH, and HH collected the clinical and imaging data. DY, QL, and LD analyzed the data. YiL, QL, DY, DL, LD, and WW co-wrote the manuscript. All authors contributed to the article and approved the submitted version.

## Funding

This study was supported by Hunan Provincial Natural Science Foundation (2022JJ30890) and Natural Science Foundation of Changsha City (No. kq2208363).

## Conflict of interest

The authors declare that the research was conducted in the absence of any commercial or financial relationships that could be construed as a potential conflict of interest.

## Publisher’s note

All claims expressed in this article are solely those of the authors and do not necessarily represent those of their affiliated organizations, or those of the publisher, the editors and the reviewers. Any product that may be evaluated in this article, or claim that may be made by its manufacturer, is not guaranteed or endorsed by the publisher.

## Footnotes

^1^ http://dbm.neuro.uni-jena.de/cat/

^2^ http://www.fil.ion.ucl.ac.uk/spm
